# Development and Application Prospects of Biomass-Based Organic Binders for Pellets Compared with Bentonite

**DOI:** 10.3390/ma18194553

**Published:** 2025-09-30

**Authors:** Yu Liu, Wenguo Liu, Zile Peng, Jingsong Wang, Qingguo Xue, Haibin Zuo

**Affiliations:** State Key Laboratory of Advanced Metallurgy, University of Science and Technology Beijing, Beijing 100083, China

**Keywords:** biomass, binder, pellet, lignin, cellulose, starch

## Abstract

With the low-carbon transformation of the steel industry, using low-carbon raw materials is one of the important ways to achieve the “dual carbon” goals. Pellets have great physical and chemical properties as low-carbon furnace materials, which can significantly reduce blast furnace carbon emissions, exhibiting favorable overall environmental benefits. Increasing their proportion in the furnace is one of the important measures the steel industry can take to reduce carbon emissions. Binders play a critical role in the pelletizing process, and their properties directly influence pellet quality, thereby affecting the subsequent blast furnace smelting process. Compared with traditional bentonite, organic binders have become a potential alternative material due to their environmental friendliness, renewability, and ability to significantly reduce silica and alumina impurities in pellets while improving the iron grade. This work systematically elucidates the mechanism of organic binders, which primarily rely on the chemical adsorption of carboxyl groups and the hydrogen bonding of hydroxyl groups to enhance pellet strength, and then provides three typical examples of organic binders: lignosulfonate, carboxymethyl cellulose (CMC), and carboxymethyl starch (CMS). The common characteristic of these organic binders is that they are derived from renewable biomass through chemical modification, which is a derivative of biomass with renewable and abundant resources. However, the main problem with organic binders is that they burn and decompose at high temperatures. Current research has achieved technological breakthroughs in pellet quality by combining LD sludge, low-iron oxides, and nano-CaCO_3_, including improved iron grade, reduced reduction swelling index (RSI), and enhanced preheating/roasting strength. Future studies should focus on optimizing the molecular structure of organic binders by increasing the degree of substitution of functional groups and the overall degree of polymerization. This approach aims to replace traditional bentonite while exploring applications of composite industrial solid wastes, effectively addressing the high-temperature strength loss issues in organic binders and providing strong support for the steel industry to achieve the green and low-carbon goals.

## 1. Introduction

Iron ore is an indispensable raw material for the steel industry. Its demand has been increasing annually with the development of the Chinese steel industry [1]. This industry has an unavoidable responsibility to reduce carbon emissions under the “dual carbon” policy [2]. Compared with other raw materials, pellets have better physicochemical properties, including uniform particle size, high iron content, better high-temperature metallurgical performance, and superior low-carbon effects [3]. It is now an important component of blast furnace burden material in China. Therefore, adjusting the burden material structure and increasing the proportion of pellets in the furnace are some of the ways the steel industry can reduce carbon emissions [4]. However, China has a high proportion of low-grade ore, and there are significant differences in the quality of pellets compared with overseas.

Pelletization typically involves mixing iron ore with binders and then adding water as a wetting agent to adjust the moisture content, promoting particle adhesion, and rolling to form uniformly sized green pellets. After that, they are dried and sintered to produce pellets with sufficient strength. As a critical component, binders increase the formation rate of pellets, enhance the strength of green pellets, and improve high-temperature metallurgical performance, which plays a foundational role in metal smelting processes [5]. In pellet production, binders enhance raw material hydrophilicity to strengthen interparticle interactions, increase the compressive strength of pellets, and ensure iron ore utilization efficiency. Additionally, binders can optimize induration and reduce the porosity caused by structural deformation, thereby improving pellet compressive strength, increasing reduction efficiency, and decreasing the reduction swelling index [6,7,8]. Ultimately, this improves pellet utilization efficiency and reduces the cost of smelting. Their wide range of sources also provides a stable and continuous supply for production needs.

Binders are classified into inorganic and organic binders. Inorganic binders mainly include bentonite, lime (CaO) and hydrated lime (Ca(OH)_2_), cement, and fly ash [9]. Bentonite is the most used inorganic binder in pellet production, and it has good hydrophilicity and water absorption. It can be fully dispersed between mineral particles after wetting, allowing for higher moisture content and improving pelletizing rate. Bentonite can be classified into sodium-activated bentonite and calcium bentonite. The former has a higher capacity of water absorption and stronger dispersion in particles, while the latter typically has lower dispersion and pellet strength [10]. However, the main components of bentonite are Al_2_O_3_ (15–25%) and SiO_2_ (60–70%), which increase the gangue content of the pellet and lead to a higher slag amount [11]. According to production experience, for every 1% bentonite added, the iron grade of the pellet decreases by approximately 0.6 wt.% and the iron content decreases by approximately 7 kg/ton. For every 1% increase in SiO_2_ content in the pellet, the cost of steelmaking increases by 4~7 USD/ton [12].

Organic binders are usually natural or synthetic polymers with high molecular weight and better hydrophilicity. They also have high viscosity in solution [13] and strong chemical interaction and bonding properties with iron ore, and they are used in lower quantities during pelletization. Unlike traditional inorganic binders, organic binders are primarily composed of C, H, and O elements. During induration, they complete combustion decomposition, avoiding the introduction of impurities such as silicon and aluminum [8], and increasing the porosity and reducibility of pellets [14]. Currently, there are many studies investigating the impact of organic binders on pellet performance.

Claremboux et al. [15] classified organic binders into three groups by binding mechanism: (1) Adsorptive binders—these binders adhere to particle surfaces via a hydroxyl (-OH) or carboxyl group (-COOH) and form bridging structures (e.g., starch, cellulose, lignosulfonate). (2) Chemically bonded binders—these undergo chemical reactions to form a cementitious compound, such as the reactions of lime and several polysaccharide compounds (e.g., molasses and dextrin) to form calcium saccharate cements [16] and epoxy [17]. (3) Dispersive binders—these binders help prevent the particles from agglomerating too rapidly [18], promote the formation of bonding structures in ultrafine material [19], and significantly improve the pelletization performance of ultrafine material. The advantage of organic binders is that they reduce the SiO_2_ content in pellets, ultimately reducing the blast furnace slag amount. Additionally, they burn at high temperatures and leave pores in the pellet, which enhances reduction performance. For example, Lyons et al. [20] replaced bentonite with organic binders, increasing pellet porosity by 29.1% and the reduction performance by 16.7%. Organic binders have very widespread global applications. Peridur [21] is a carboxymethyl cellulose-based binder widely used in South America, Funa [22] is a modified humic acid binder (MHA) developed and applied in China, and polyacrylamide (PAM) binders have also been applied industrially, such as in the United States and Russia. However, organic binders also have problems such as low strength and poor wear resistance, which lead to significant dust pollution during pellet production. Additionally, organic binders have higher costs and are significantly influenced by the type of iron ore.

Among the three types of organic binders, adsorptive organic binders have been applied the most widely, as their special bonding mechanism can effectively increase the strength of green pellets. Among these, lignosulfonate (LS), carboxymethyl cellulose (CMC), and carboxymethyl starch (CMS) are more special types of adsorptive organic binders, which are derived from renewable biomass through chemical modification, belonging to biomass derivatives. The value of these binders is their environmental friendliness and sustainability. Biomass is a clean, renewable, and sustainable energy resource that is widely available in nature and is the fourth largest energy resource after coal, natural gas, and oil [23]. China produces approximately 1.3 billion tons of agricultural and forestry waste annually, with the total amount of usable biomass resources equivalent to approximately 460 million tons of standard coal [24]. Biomass can be considered a carbon-neutral renewable energy source because it releases carbon dioxide during combustion, which is absorbed from the surrounding environment during its growth [25].

Cellulose is the most abundant biopolymer in woody biomass, followed by lignin. These two are the primary components of plant cell walls, which represent the largest and second-largest renewable natural carbon sources, respectively [26]. In the papermaking industry, cellulose is the main component of paper. During the pulping process, lignin is converted into lignosulfonate and discarded as waste in black liquor [27]. Starch is one of the most abundant polysaccharides in nature, which has a wide range of applications in food, industry, and medicine [28]. LS, CMC, and CMS, as biomass derivatives, all show great potential as organic binders for pellets. However, current research on the application effects, existing problems, and solution strategies of these biomass-based organic binders in pellets has not yet been systematically and comprehensively reviewed and analyzed. Based on this, this work aims to provide a systematic review of the application of biomass-based organic binders in pellet production, summarize their advantages and disadvantages, explore effective improvement methods, and offer insights and references for the better application of organic binders in the preparation of low-carbon furnace materials in the steel industry.

## 2. The Binding Mechanism of Biomass-Based Binder in Pellets and Its Application Potential

**Figure 1:**
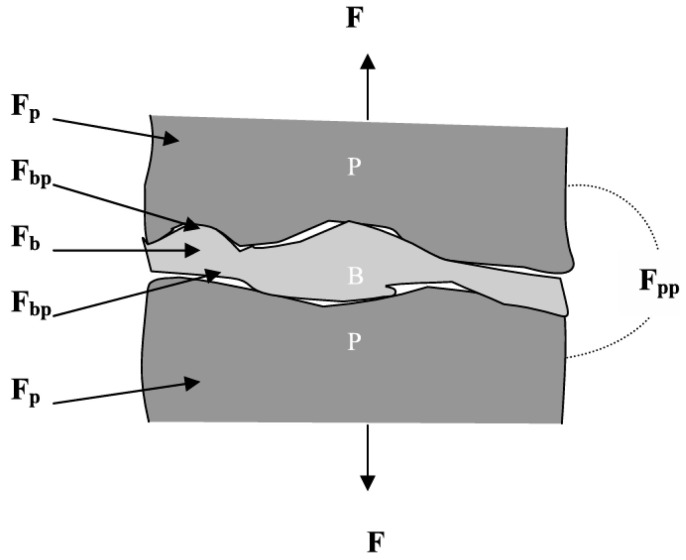
Binding model and interaction forces inside particle–binder systems (where P is mineral particles, B is binder, F is force) [29].

Figure 1 shows a model of the mineral particle–binder system within a pellet. This system consists of two phases and an interface: binder B, mineral particles P, and the interface BP between B and P. Four kinds of interaction forces exist in this system [29]: (1) Binder cohesive force F_b_—F_b_ is the sum of the interaction forces between molecules and atoms within the binder, representing the mechanical properties of the binder itself. (2) Ore cohesive force F_p_—F_p_ is the sum of the intermolecular and ionic interactions within the mineral particles, representing the mechanical properties of the ore particles. (3) Adhesive force at the interface F_bp_—F_bp_ is the total of van der Waals, electrostatic, magnetic, hydrogen bonding, chemical bonding, and viscous forces between the binder and ore particles. (4) Interaction force between ore particles F_pp_—F_pp_ is the sum of the intermolecular forces between mineral particles, including van der Waals forces, electrostatic forces, magnetic forces, mechanical interlocking forces, and capillary forces (when water is present).

**Figure 2:**
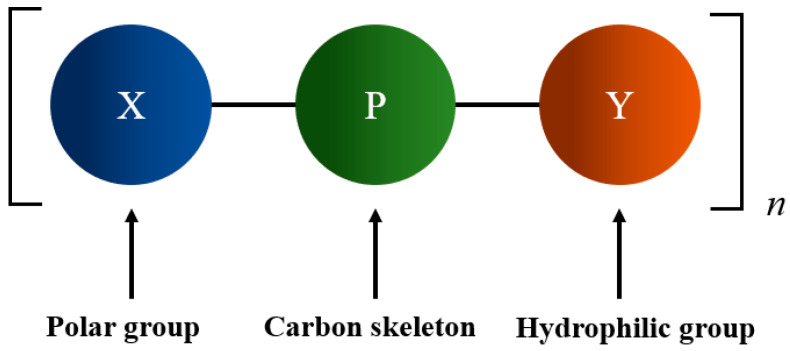
Molecular structure model of an ideal organic binder [29].

Qiu et al. [29] first proposed an ideal organic binder model (Figure 2). In this model, X represents a polar group that can strongly adhere to the surface of ore particles. Y represents a hydrophilic group that improves the hydrophilicity of ore particles. P represents an organic chain framework that stabilizes the molecular structure, prevents breakage, and provides good bonding properties. The electronegativity of functional groups served as the theoretical basis for selecting polar groups in binders. Electronegativity values of different polar groups were calculated according to HMO (Hückel molecular orbital method) indices, which indicate that the carboxyl group is an ideal polar group, the hydroxyl group is a relatively ideal hydrophilic polar group, and a network or benzene ring structure is a relatively ideal organic chain framework [30]. Polar groups enhance the adhesive force through adsorption with the surface of ore particles; hydrophilic groups improve the hydrophilicity of ore particles, promoting contact between particles and the binder; and organic chains provide structural support for the cohesive force of the binder. Research indicates that the bonding strength of the ore particle–binder system depends on the cohesive force (F_b_) of the binder and the adhesive force (F_bp_) at the interface between the binder and ore particles [31,32,33].

### 2.1. The Adhesion Between the Binder and the Surface of Iron Ore

**Figure 3:**
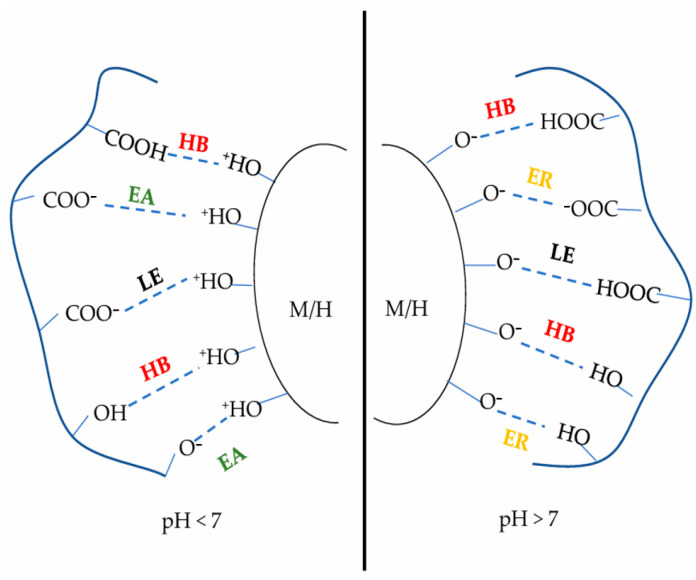
Surface interactions between organic binders and magnetite (M)/hematite (H) minerals. HB: hydrogen bond; EA: electrostatic attraction; ER: electrostatic repulsion; LE: ligand exchange [13].

The adhesive force between the adhesive and ore particles is usually achieved by the interaction between the hydroxyl or carboxyl groups and the ore particles [13]. The mineral surface is hydroxylated upon contact with water and adsorbs H^+^ from the solution to form Fe-OH^2+^ [34]. Meanwhile, the carboxyl groups of the organic binder dissociate to form -COO^−^, and both of them interact through electrostatic attraction, promoting the formation of a HO-P-OC-O-Fe coordination bond structure between the carboxylic acid oxygen atoms and the iron atoms on the surface of the iron ore particles, thus increasing the bonding strength [35]. Under alkaline conditions, the hydroxyl groups on the surface of ore particles deprotonate to form negatively charged Fe-O^−^, while the carboxyl groups further combine with OH^-^ to form more COO^−^, and the electrostatic repulsion reduces the adhesive capacity [36]. The interaction between the binder and the surface of ore particles is shown in Figure 3. Although electrostatic repulsion affects adsorption on the ore surface negatively, organic binders can form strong bonding forces through molecular networks, thus enhancing the bonding strength between ore particles.

### 2.2. The Cohesion of the Binder

Cohesive force is the interaction force between binder molecules, which is determined by the polymerization degree, chemical structure, crosslinking degree, and branching degree [37]. Qiu et al. [29] suggested that polymer molecules must possess a strong organic chain structure (e.g., aromatic rings in lignin or β-1,4 glucosidic bonds in cellulose) and a high degree of polymerization to exhibit greater cohesive strength. As this degree increases, the interactions of the interchain become stronger, leading to longer branched chains that intertwine [13], enhancing van der Waals forces, hydrogen bonds, and other interactions of the interchain. The entanglement phenomenon of this chain directly results in an increase in the viscosity of the binder solution, which can effectively control moisture transport within the pellet and improve its pelletization capability within an effective timeframe [38]. Binders with high viscosity tend to slow down the growth rate of pellets, promoting a more uniform distribution of moisture and reducing localized over-wetting or over-drying. This helps reduce defects during pelletization and improve pellet strength. Therefore, appropriately increasing the viscosity of organic binders is an important method to effectively enhance pellet strength and pelletization efficiency [7].

### 2.3. The Application Potential of Biomass-Based Binder

In the field of metallurgical pelletizing, biomass-based organic binders, such as LS, CMC, and CMS, are showing great potential for application. Many biomass-based organic binders are emerging as research into binders develops (Table 1). These green binders can effectively replace some inorganic binders like bentonite after modification, which are from plant fibers, wood, or starch, and combine excellent binding performance with environmental friendliness. These binders leave extremely low ash residues after high-temperature decomposition, helping reduce impurity accumulation in blast furnace smelting processes. Additionally, their biodegradable properties conform to the low-carbon transformation requirements of the metallurgical industry. With future technological breakthroughs, biomass-based organic binders will play an even more critical role in enhancing pellet performance, reducing energy consumption, and promoting the development of green processes.

## 3. Application Characteristics of Some Composite Binders as Pellet Additives

### 3.1. Lignosulfonate

#### 3.1.1. Sources and Structure of Lignosulfonate

Lignin is a renewable polymer that is widely present in nature, with a content of approximately 15–40% of biomass [51]. Of the 50–70 million tons of lignin produced each year, only 1–2% is used for other production [52], indicating that lignin is an underused material [53]. Lignosulfonates are byproducts of the sulfite pulping process of wood [54], during which the lignin network is decomposed and sulfonic acid groups are introduced. Currently, lignosulfonates account for 90% of the total commercial market for lignin, with an annual global production of approximately 1.8 million tons [55,56,57].

**Figure 4:**
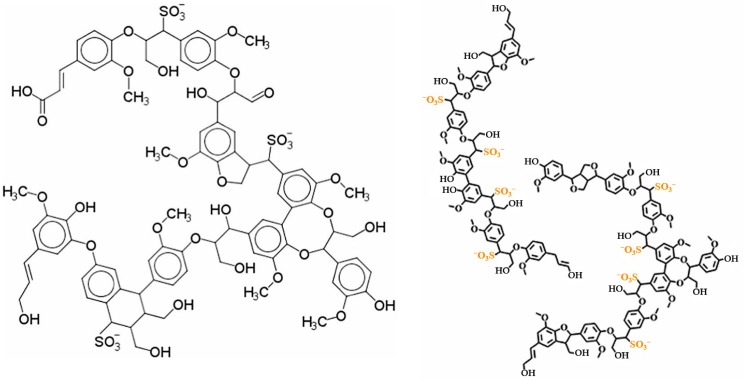
Simplified structure of lignosulfonates according to Kun et al. [60] (**left**) and Fiorani et al. [61] (**right**).

Lignosulfonates can be characterized as randomly branched polyaromatic polyelectrolytes, with their hydrophilicity primarily attributed to the presence of anionic sulfonate groups (-SO_3_^−^), anionic carboxylate groups (-COO^−^), and hydroxy groups (-OH) under alkaline conditions [58]. The counterion is often a remnant from the pulping process, such as Na, Ca, and Mg, which may also determine the physicochemical properties of lignosulfonates through other ways, such as affecting polymer conformation [59]. Figure 4 shows two typical examples of lignin sulfonate structures.

#### 3.1.2. Interaction Between Lignosulfonate and Minerals

Lignosulfonates contain a large number of hydrophilic sulfonic acid groups and electrically active methoxyphenol groups. The unpaired electron pairs of oxygen and sulfur atoms in these groups can serve as coordination sites for metal ions, meaning that these negatively charged functional groups can adhere to positively charged mineral surfaces through electrostatic attraction [62].

The degree of sulfonation (DS) is defined as the millimolar content of sulfonic acid per gram of lignosulfonate [63], which affects its various physicochemical properties, such as water solubility [64] and dispersing performance [65]. The sulfonation degree of lignin sulfonate fluctuates within the range of approximately 0.4–0.7, providing sufficient hydrophilic groups (-SO_3_^−^) [66]. This guarantees that it can quickly diffuse to the surface of iron ore and has good wettability, making it possible to adhere more fully to the surface of iron ore within an effective time, thereby improving the pelletization rate. Lignosulfonates have a broad molecular weight distribution (M_w_ = 5000–60,000 g/mol), which results in a high degree of polymerization, forming longer branched chains that intertwine with each other, and ensuring sufficient cohesive force [67].

#### 3.1.3. Process Characteristics and Application Effect of Lignosulfonate

**Figure 5:**
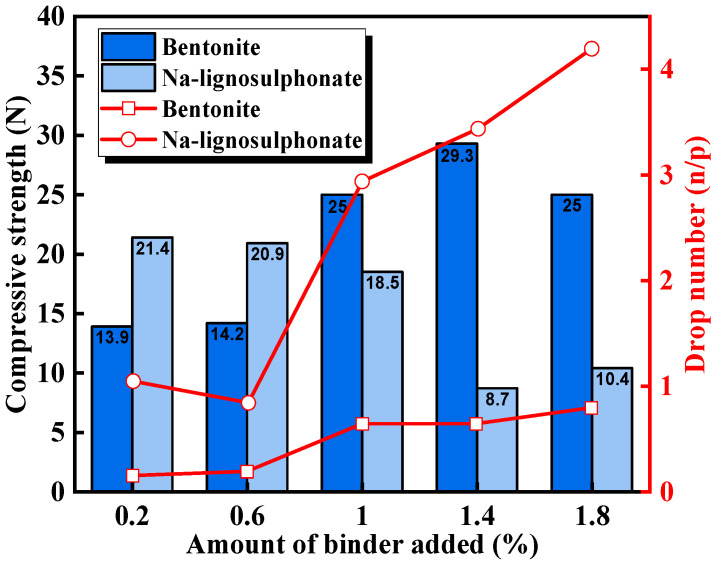
The effect of different binders on the drop strength and compressive strength of green pellets [40].

Biomass-based organic binders do not contain inorganic substances such as SiO_2_ and Al_2_O_3_. These organic binders are completely burned and decomposed during induration, enhancing the iron content of the pellets, reducing the accumulation of harmful substances in the furnace, and decreasing slag volume. The addition of binders is a key factor in determining pellet performance. The experimental results for limonite by Fang et al. are shown in Figure 5 [40]. The results indicated that lignosulfonate achieved the basic requirements for green pellet strength at low content, and only 0.2% sodium lignosulfonate (Na-LS) achieved optimal green pellet compressive strength. Additionally, the drop number of green pellets containing sodium lignosulfonate was significantly better than that of bentonite pellets.

To overcome the loss of strength of organic binders at high temperatures, researchers have proposed adding materials that contain low iron oxides to compensate for it, such as grinding slag (containing FeO and Fe_3_O_4_) and LD sludge (containing metallic iron and FeO). The principle behind this is that these low-iron oxides can oxidize to Fe_2_O_3_ at lower temperatures and form diffusion bonds within the pellets, thus providing additional bonding strength in the temperature range where the organic binder fails. Pal et al. [39] effectively suppressed the sudden decrease in strength observed around 350 °C by adding calcium lignosulfonate (Ca-LS) and 3% LD sludge (Table 2) Ammasi et al. [42] provided another example by using Na-LS and copper smelting slag (mainly FeO) as pellet binders. Their results indicated that the enhanced pellet strength resulted from improved diffusion and recrystallisation bonding.

**Figure 6:**
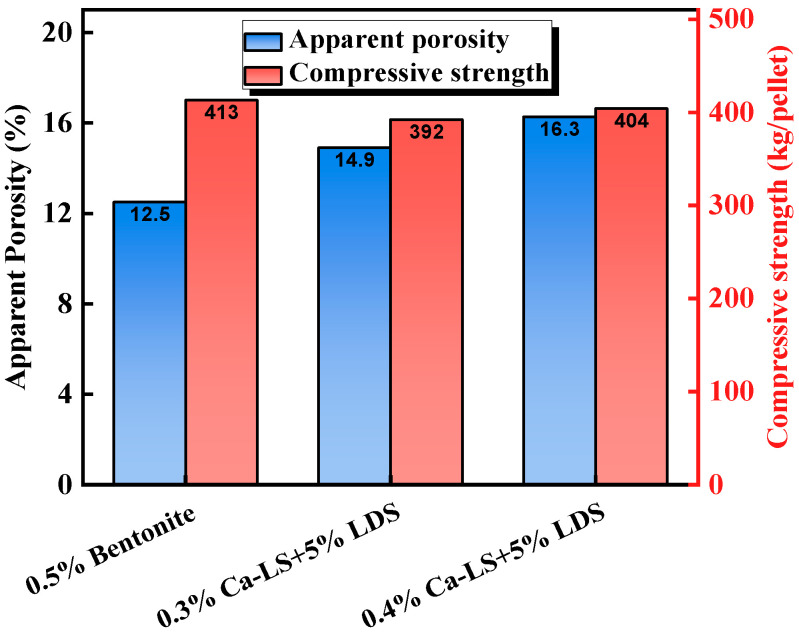
Physical properties of pellets after heating at 1250 °C [39].

**Figure 7:**
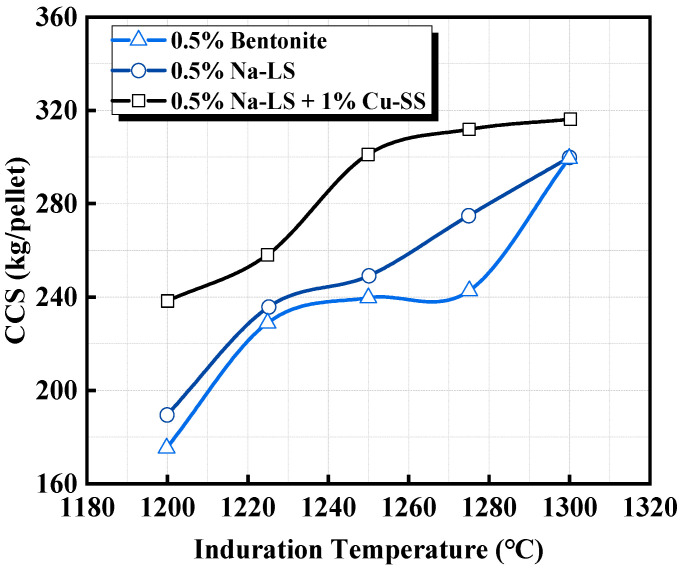
Comparison of CCS for three different indurated pellets [42].

The optimized biomass-based binder pellets exhibit physicochemical properties after induration that are comparable with or even better than those of traditional bentonite pellets. Figure 6 and Table 3 display the experimental results of Pal et al. [39]. The study indicated that Ca-LS pellets with 5% LD sludge addition had a comparable compressive strength with bentonite pellets after induration, while the apparent porosity was improved, which was beneficial for gas diffusion during the reduction process. In metallurgical performance, Ca-LS and LD sludge pellets had a higher iron grade, with a comparable reducibility to bentonite pellets, while the low-temperature reduction degradation index (RDI) and reduction swelling index (RSI) were significantly lower than those of bentonite pellets, which is a prominent advantage. Ammasi et al. [42] showed similar results in their study of Na-LS and Cu-SS pellets, indicating that the combination of 0.5% Na-LS and 1% Cu-SS provided the best performance (Figure 7, Table 3). The addition of only 0.5% Na-LS resulted in a higher RDI and RSI, attributed to strength loss and density loss caused by burning at 600–900 °C, while the addition of Cu-SS effectively solved this issue.

**Figure 8:**
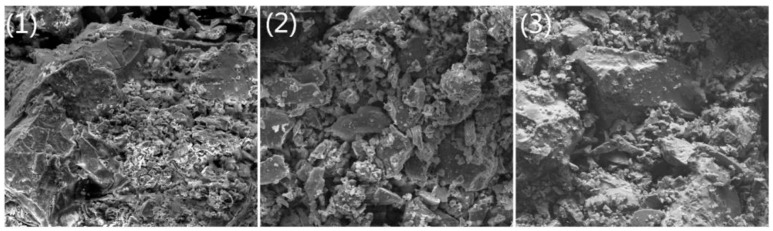
SEM images of sintered pellets: (**1**) 0.5% LS, (**2**) 2% bentonite, and (**3**) 0.5% LS + 1% CaCO_3_ [43].

Research on the microstructure of roasted pellets revealed the mechanism of action of organic binders and additives. The SEM analysis by Zhou et al. [43] is shown in Figure 8, indicating that particles in lignosulfonate pellets exhibit a highly dispersed state with few contact points, and the structure between gangue and iron oxide particles was loose. However, the addition of CaCO_3_ promoted particle fusion, aggregation, and agglomeration, resulting in a more compact structure. This promoted bridging, bonding, and recrystallization processes between magnetite particles [68], increasing the compressive strength of the sintered pellets.

### 3.2. Carboxymethyl Cellulose (CMC)

#### 3.2.1. Sources and Structure of CMC

**Figure 9:**
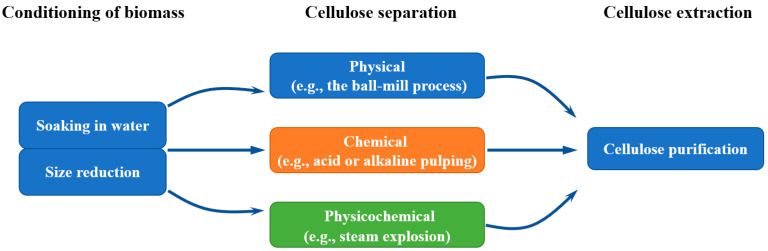
Three basic processes in the extraction of cellulose from lignocellulosic biomass.

Cellulose is the most abundant natural biomass on Earth and a crucial component of plant cell walls. It is sustainable and biodegradable, with approximately 10^11^–10^12^ tons of biomass capable of producing synthetic cellulose annually [69], and with the global production of cellulose reaching approximately 192 million tons per year [70]. Recent studies have shown that cellulose can be extracted from various plants, such as corn stalk, sugarcane bagasse, cocoa husks, rice husks, coconut fibers, and other agricultural wastes [71,72,73,74,75]. The extraction process is shown in Figure 9. As the concern for environmental issues continuously increases, cellulose and its derivatives are gaining more attention. These cellulose derivatives can be classified into cellulose ethers or cellulose esters based on the reagents used during the chemical modification. Carboxymethyl cellulose (CMC) is one of the cellulose derivatives and belongs to cellulose ethers [76,77,78].

**Figure 10:**
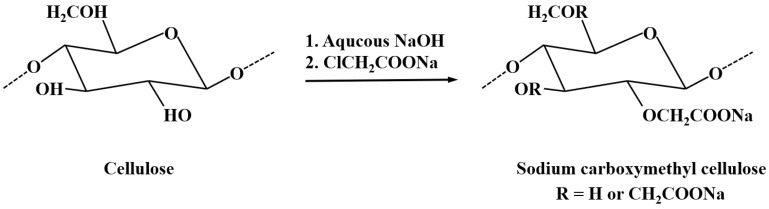
The reaction scheme for the preparation of CMC [81].

CMC is water-soluble and is known for its high viscosity, biocompatibility, and thermal stability [79]. It is commonly used as a thickener, stabilizer, emulsifier, and binder, and has become a key material in many industries, including food, pharmaceuticals, metallurgy, textiles, and oil drilling [80]. Carboxymethyl cellulose is prepared in the form of a sodium salt through the reaction of alkaline cellulose with chloroacetic acid (ClCH_2_COOH) or its sodium salt, and the reaction scheme for the preparation of CMC is shown in Figure 10.

(1) $${\left[C_{6}H_{7}O_{2}{\left(OH\right)}_{3}\right]}_{n}+nxNaOH+nxClCH_{2}COONa\to \;{\left[C_{6}H_{7}O_{2}{\left(OH\right)}_{3-x}{\left(OCH_{2}COONa\right)}_{x}\right]}_{n}+nxNaCl+nxH_{2}O$$

#### 3.2.2. Interaction Between CMC and Minerals

CMC contains many hydroxyl and carboxyl groups in its molecules, which can adhere to metal cations (such as Fe^2+^ and Fe^3+^) on the surface of iron ore and form a stable structure. Under humid conditions, CMC molecules adhere to ore particles through chemical bonds, electrostatic attraction, van der Waals forces, and hydrogen bonds [82]. Under dry conditions, CMC adheres to surfaces by forming chemical bonds with Fe^2+^/Fe^3+^ through oxygen atoms (-OH, -CH_2_COOH, or -CH_2_OCH_2_) [83]. Additionally, CMC has excellent water solubility and viscosity, which can uniformly fill interparticle gaps during pellet preparation, enhancing the strength of the pellets.

In the synthesis of CMC, the degree of substitution (DS) is defined as the average number of hydrogen atoms on the hydroxyl group that are replaced by carboxymethyl groups in each dehydrated glucose unit [71], with values typically in the range of 0 to 3 [84,85]. DS plays a vital role in the properties of CMC, including solubility, emulsibility, thickening, acid resistance, viscosity, stability, and salt tolerance properties [86]. G.M. Yang et al. [87] indicated that CMC with higher DP or DS has better adhesive properties. A higher DS usually means that more hydroxyl groups on the cellulose molecular chains are replaced by hydrophilic carboxymethyl groups, which not only significantly improves the solubility of CMC in water, making it easier to form a uniform and effective binder solution, but also reduces the interaction between the binder and various ions in the solution. On the other hand, DP is a key parameter for measuring molecular weight: the higher the value, the longer the molecular chain [88]. Long molecular chains provide more entanglement points and contact sites with mineral particle surfaces in solution, generating stronger intermolecular forces and forming a denser and more continuous adhesive structure, thereby significantly enhancing the bonding force between ore particles.

#### 3.2.3. Process Characteristics and Application Effect of CMC

**Figure 11:**
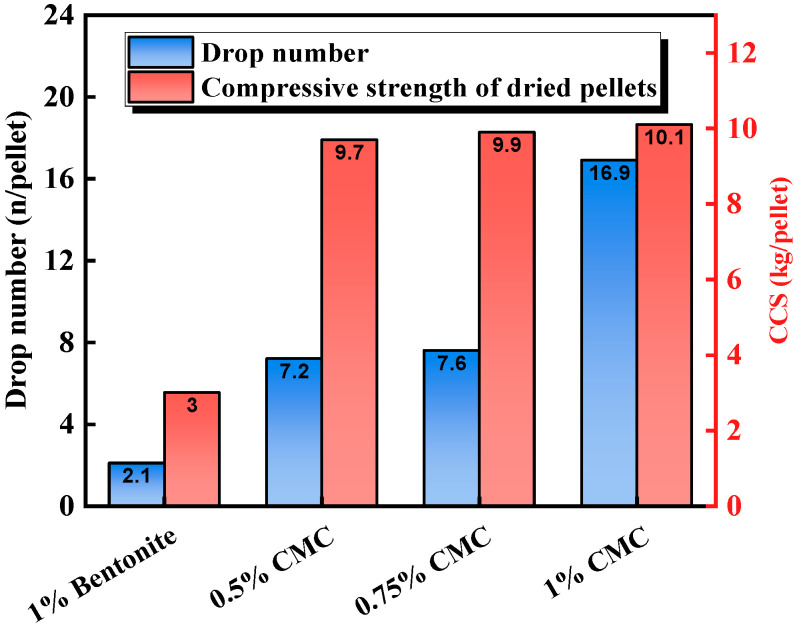
The drop strength and the compressive strength of dried pellets of CMC [44].

In the study of organic binders as alternatives to bentonite, modified sugars such as CMC have been extensively researched due to their potential to enhance the strength of green and dry pellets [89]. However, it may be difficult to meet the strength requirements of pellets after induration with the use of only organic binders. Parathodiel et al. [44] studied the drawbacks of CMC as a pellet binder at high temperatures in terms of strength loss, and the experimental results are shown in Figure 11. CMC pellets showed a higher drop strength and dry ball compressive strength than bentonite pellets, exhibiting excellent green ball strength performance. However, Parathodiel found that pellets containing only CMC had a lower strength after induration, with this strength loss attributed to CMC burning at approximately 390 °C [90], making it unable to provide effective bonding at high temperatures.

To overcome the insufficient strength of organic binders at high temperatures while avoiding the introduction of harmful impurities, researchers proposed a strategy of adding specific fluxing materials [91]. Lu et al. [47] used nano-CaCO_3_ and CMC as a composite binder. Compared with single CMC pellets, CCMC (nano-CaCO_3_ and CMC) significantly improved the preheating pellet strength and calcination pellet strength. They revealed the micro-bonding mechanism, with nano-CaCO_3_ particles tightly covering the CMC surface, promoting the formation of more molten phases. These molten phases effectively filled the pores between particles, facilitating the bridging, bonding, and recrystallization processes between hematite particles [92]. The oxide bridging and bonding between hematite particles, and the formation of additional slag phases, collectively reduce the porosity of the roasted pellet and increase its compressive strength.

**Figure 12:**
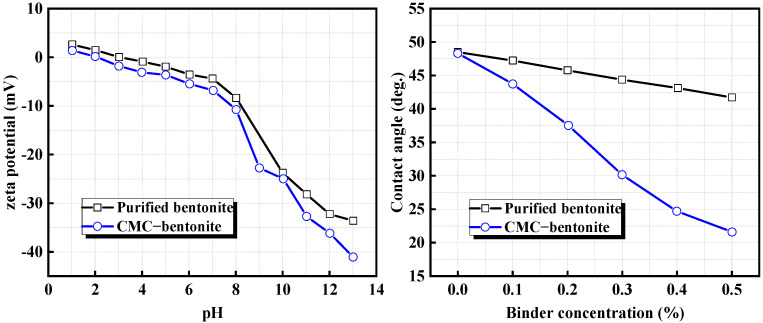
Effect of binder type and content on zeta potential and surface contact angle [46].

The composite of CMC with traditional bentonite is also effective in enhancing the performance of the binder. Li et al. [46] studied this in depth; they measured the relationship between the pH value and zeta potential of different binders, as well as the effect of binder content on the surface contact angle of iron ore. Figure 12 shows the experimental results, indicating that the CMC–bentonite composite binder had a lower zeta potential and exhibited stronger water absorption and swelling rates. As the content of the composite binder increased, the contact angle on the surface of the iron concentrate gradually decreased, indicating that the hydrophilicity of the magnetite surface was significantly improved, which is beneficial for nucleation and growth during the pelletization process. The addition of CMC promotes further dissociation of the unpeeled layer of bentonite, resulting in finer particles, larger specific surface area, and more uniform dispersion. Additionally, CMC molecules enhance interparticle bonding strength and capillary forces generated by liquid bridges through adhesion, thereby providing a stronger cohesive force during the pelletization process.

### 3.3. Carboxymethyl Starch

#### 3.3.1. Sources and Structure of Carboxymethyl Starch

Starch is a renewable, low-cost, and abundant natural biomass, which is the main storage carbohydrate of plants [93]. Starch consists of two types of glucose polymers: straight- and branched-chain starch [94]. Products made from starch exhibit renewability, biodegradability, and environmental friendliness [95]. However, natural starch has some drawbacks, such as poor flowability, insolubility in cold water, and difficulty in controlling viscosity, which significantly limit its application [96].

CMS is one of the simplest derivatives of starch, which contains abundant carboxyl and hydroxyl groups on its chain backbone [97]. It is a water-soluble polysaccharide and widely used in numerous industrial fields [98]. Among these, corn starch and potato starch are commonly used in industrial production due to their high content of branched-chain starch (approximately 80–90%) and good reactivity. CMS is produced through a two-step reaction between starch (St-OH) and sodium chloroacetate in a sodium hydroxide environment. The first step involves the alkalization of starch, followed by an etherification reaction [99]:

(2) $$StOH+NaOH\to StNa^{+}+H_{2}O$$

(3) $$StONa^{+}+ClCH_{2}COONa\to StOOCH_{2}COONa+NaCl$$

where St is the glucose residue (C_6_H_9_O_7_) in starch.

CMS is composed of glucose units linked by α-1,4 glycosidic bonds [100], forming a straight-chain structure. The hydroxyl group at the C6 position of each glucose unit is replaced by a carboxymethyl group (-CH_2_COO^−^), resulting in an anionic polymer [101]. CMC is characterized by water solubility, high viscosity, biodegradability, and adhesive properties. Currently, many studies are investigating its effects as a binder on the performance of pellets.

#### 3.3.2. Interaction Between CMS and Minerals

CMS primarily interacts with the surface of iron ore through the carboxymethyl and hydroxyl groups. Chemical adhesion is the dominant mechanism, with the carboxyl group forming chemical bonds with the Fe-OH groups on the ore surface. Additionally, the hydroxyl group assists in the adhesion through hydrogen bonding, further enhancing the bond strength. Lu et al. [48] used AFM imaging to indicate that CMS molecules form a continuous, thick film structure on the ore surface, effectively filling the gaps between iron ore particles and covering the mineral surface. Furthermore, Li et al. [102] noted that after the addition of CMS, the contact angle on the surface of magnetite concentrate decreased from 48° to 5°, enhancing the hydrophilicity of the mineral particle surface. Additionally, it increases the viscosity of liquid bridges, thereby enhancing interfacial energy, capillary attraction energy, and viscous interaction energy between particles, significantly improving pellet strength. The CMS is a spatial network structure, making it an ideal organic binder chain structure for pellets. This structure ensures good dispersion of the binder, enabling uniform distribution within the pellets, facilitating uniform steam evaporation, and improving the thermal stability of the pellets.

#### 3.3.3. Process Characteristics and Application Effect of CMS

**Figure 13:**
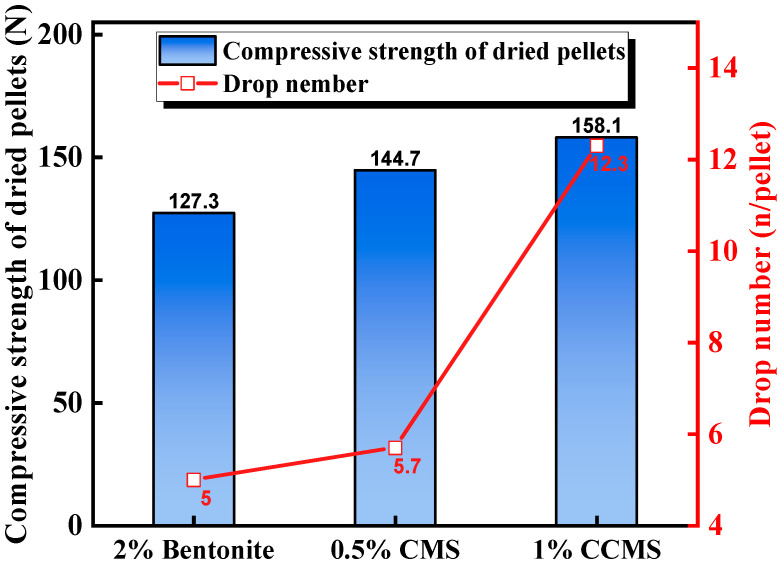
The effect of binder type on the drop number of green pellets and the compressive strength of dried pellets [50].

**Figure 14:**
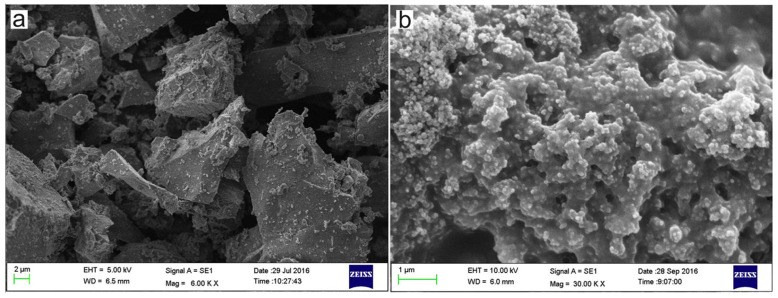
SEM micrographs of (**a**) pure magnetite concentrate and (**b**) CMS + nano-CaCO_3_ [49].

As mentioned above, the characteristic of organic binders is that they can impart good performance to green pellets at low content. However, their drawback is that the strength of the pellets is insufficient after preheating and sintering, which may be related to the high porosity and low glass phase content in the pellets. Lu et al. [50] systematically compared the effects of bentonite, CMS, and CCMS (nano-CaCO_3_ and CMS) on pellet performance. Regarding the green pellet performance, CCMS pellets showed the highest drop number, significantly better than bentonite and CMS pellets (Figure 13). This indicated that the addition of nano-CaCO_3_ effectively enhances the bonding ability of CMS, improving the elasticity and impact resistance of the green pellets. Yuan et al. [49] provided a mechanistic explanation for this, as shown in the microstructure in Figure 14. It was found that plate-like CMS molecules and spherical nano-CaCO_3_ particles formed a unique cross-linked skeletal structure, which served as a support network between CMS molecules, significantly improving the bonding performance of the composite binder.

**Figure 15:**
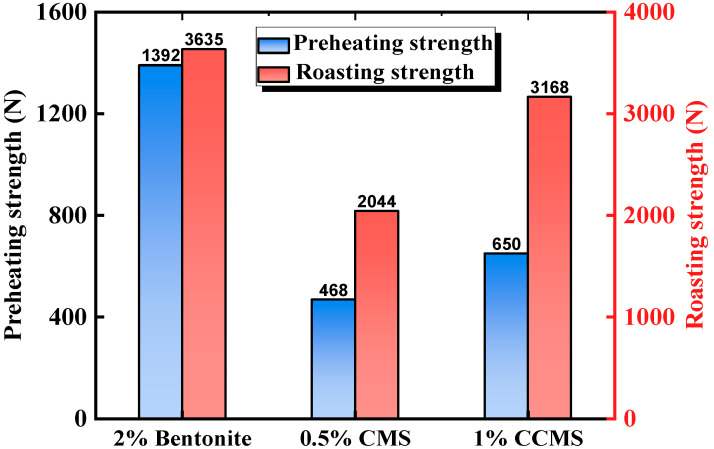
The effect of binder type on preheating strength and sintering strength [50].

**Figure 16:**
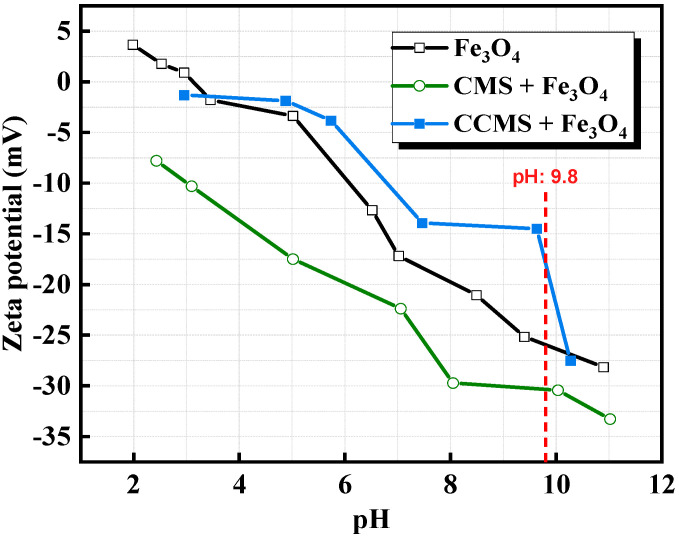
Zeta potential of magnetite concentrate before and after interaction with binders [49].

However, CMS pellets exhibited significant strength loss at high temperatures. Figure 15 shows the experimental results of Lu et al. [50], where the preheating strength was lower than the minimum standard (500 N). However, the preheating strength of CCMS pellets was significantly improved, attributed to the dual role of nano-CaCO_3_ in the pellets. First, nano-CaCO_3_ acted as a fluxing agent, promoting the formation of a low-temperature melt phase during heating to compensate for the strength loss. Second, the research of Yuan et al. [49] indicated that although magnetite, CMS, and nano-CaCO_3_ all carried negative charges at pH = 9.8, theoretically resulting in electrostatic repulsion, experimental observations showed a positive shift in the zeta potential. This confirmed that chemical interactions and hydrogen bonding forces dominated the bonding between the three components, enabling nano-CaCO_3_ to tightly connect CMS molecules with mineral particles and enhance structural stability (Figure 16).

During induration, the results of Lu further indicated that the roasting strength of CCMS pellets was also significantly higher than that of CMS pellets and met industrial standards. During the high-temperature induration, nano-CaCO_3_ promoted the filling of pores with more molten phases, reduced the distance between particles, and optimized the bridging and recrystallization processes of hematite particles, thereby offsetting the negative effects of the combustion and decomposition of organic binders and improving the overall performance of the pellets.

### 3.4. Discussion

This section systematically discusses the mechanisms of action and application characteristics of three biomass-based organic binders in the pellet ore. Research indicates that these polymeric compounds derived from renewable biomass, with abundant hydrophilic functional groups, significantly enhance green pellet strength and achieve superior pellet formation performance compared with bentonite at a lower content. However, organic binders suffer from poor high-temperature thermal stability. They burn and decompose during preheating and induration, leading to a significant reduction in pellet strength. Particularly, the sharp drop in strength occurring within 300–600 °C severely limits their industrial applicability.

To overcome these limitations, researchers proposed introducing inorganic additives such as low-iron oxides to achieve strength compensation. Pal et al. [39] significantly suppressed the strength decrease of Ca-LS pellets near 350 °C by adding 3% LD sludge. The mechanism stems from the low-iron oxides oxidizing to Fe_2_O_3_ at lower temperatures and forming diffusion bonds within the pellets, thereby providing additional bonding forces. This mechanism was also supported by the research of Ammasi [42]. Furthermore, optimized composite binders, such as 5% LD sludge + Ca-LS or 1% Cu-SS + 0.5% Na-LS, achieved a compressive strength comparable with or exceeding that of bentonite pellets after induration. These formulations also increased the iron grade by 1–2% and reduced reduction expansion by over 30%.

For CMC and CMS, researchers effectively compensated for high-temperature strength loss by introducing nano-CaCO_3_. Experimental data indicate that the compressive strength of their roasted pellets significantly surpassed that of single organic binders, meeting or even exceeding the standards required by industrial processes. Microstructural analysis further confirms that the addition of nano-CaCO_3_ promotes the formation of a low-temperature melt phase and the bridging recrystallization of hematite particles, thereby enhancing bonding forces. Additionally, the CMC–bentonite composite system enhances the zeta potential and hydrophilicity, thereby optimizing liquid bridge forces and capillary action during pellet formation. This maintains excellent pellet properties while reducing material consumption. In summary, the binding strategy combining biomass-based organic binders with inorganic compounds not only effectively overcomes the high-temperature limitations of single organic binders but also demonstrates potential to replace traditional bentonite.

Additionally, the initial precursor of biomass-based organic binders (lignin, cellulose, and starch) primarily influences the final properties of the binder through the degree of substitution (DS), the degree of polymerization (DP), and the organic chain skeleton. DS and DP are key parameters affecting organic binder performance. A higher DS generally indicates that more hydroxyl groups on the molecular chain of organic binders are substituted by hydrophilic groups. This not only significantly enhances its solubility in water, facilitating the formation of a uniform and effective binder solution, but also reduces interactions between the binder and various ions present in the solution. DP serves as a key parameter for measuring the molecular weight of organic binders, with higher values indicating longer molecular chains. Longer molecular chains provide more entanglement points and contact sites with mineral particle surfaces in solution, resulting in stronger intermolecular forces and forming a denser binding structure. This significantly enhances the cohesive force between iron ore particles.

The organic chain skeleton ensures molecular structural stability and reduces breakage. For example, lignin consists of three phenylpropane units linked together through various C-O and C-C bonds, forming a complex polymeric structure. Cellulose comprises rigid linear macromolecular chains of glucose units connected by β-1,4-glycosidic bonds. Starch, mostly composed of amylopectin, features a highly branched dendritic structure formed by short glucose chains linked via α-1,6-glycosidic bonds. These complex structures provide binders with excellent bonding strength.

## 4. Conclusions

In the background of the low-carbon transformation of the steel industry, using low-carbon raw materials is one of the important ways to achieve the “dual carbon” goals. Pellets have great physical and chemical properties as low-carbon furnace materials, where increasing their proportion in the furnace is one of the important measures the steel industry can take to reduce carbon emissions. Binders play a critical role in the production of pelletized ore, with their performance directly affecting the quality of the pellets. Organic binders, as an alternative to bentonite, enhance pellet performance by chemically adsorbing and forming hydrogen bonds with Fe-OH sites on the surface of ore particles through carboxyl and hydroxyl groups. Biomass is a clean, renewable, and sustainable energy source widely available in nature. Using its derivatives in pellet production can promote low-carbon emissions reduction in the steel industry, such as LS, CMC, and CMS, which are three representative biomass-based organic binders. The characteristic of organic binders is that they can achieve good performance with minimal content. However, their drawback is insufficient pellet strength after preheating and sintering, requiring the addition of other substances to compensate for strength loss. The current application results with better performance are as follows:

1. Na-LS relies on the chemical adhesion of sulfonic acid groups but requires the addition of low-iron oxides such as LD sludge/copper slag to compensate for high-temperature strength loss, which can increase the iron grade of pellets by 1–2% and reduce the RSI by more than 30%.
2. CMC achieves a strong cohesive force through a high degree of substitution (DS > 0.8) and high polymerization degree, improving green pellet performance. However, nano-CaCO_3_ needs to be added to form a molten phase that fills pores, addressing the issue of insufficient sintering strength.
3. CMS forms a continuous thick film with iron ore through the action of polar groups. The charge compensation effect of nano-CaCO_3_ (isoelectric point pH = 9.8) overcomes electrostatic repulsion, increasing the preheating strength of pellet ore by 40%. Both preheating strength and roasting strength meet industrial standards, with the iron grade improved by 1.04%.

All three types of biomass-based organic binders require the addition of inorganic materials to address strength loss. Their advantage is that they can reduce impurities such as Si and Al in pellets, and enhance porosity and reducibility, meeting the green and low-carbon development needs of the metallurgical industry. Future research should focus on optimizing the molecular structure of organic binders, increasing the substitution degree of functional groups and the overall molecular polymerization degree, replacing traditional bentonite, and exploring the application of composite industrial solid waste materials. This will effectively address the strength loss issues of organic binders at high temperatures, providing strong support for the steel industry to achieve its green and low-carbon strategic goals.

## Figures and Tables

**Table 1:** Research and application of biomass-based organic binders.

Binders	Additives	Specific Performance	Ref.
Ca-lignosulphonate (Ca-LS)	LD sludge	Pellets containing 0.4% Ca-LS and 5% LD sludge were comparable with bentonite-added pellets.	Pal et al. [39]
Na-lignosulphonate (Na-LS)	/	The compressive strength of the preheated pellets reached 342.55 N/P at 1100 °C.	Fang et al. [40]
Na-lignosulphonate (Na-LS)	/	The drop number, compressive strength, and porosity were better than those of the bentonite-added pellets.	Tafadzwa et al. [41]
Na-lignosulphonate (Na-LS)	Copper slag, Limestone	Using only 1.0% copper slag and 0.5% Na-LS, good CCS (300 kg/pellet) at 1250 °C, the highest RI (82.5%), low RDI (18%), and low swelling index (11%) were achieved.	Ammasi et al. [42]
Na-lignosulphonate (Na-LS)	CaCO_3_	The CaCO_3_ promoted the binding ability of LS and had good compressive strength after preheating and induration.	Zhou et al. [43]
Carboxymethyl cellulose (CMC)	/	Green pellets with only CMC added exhibited excellent performance.	Parathodiel et al. [44]
Carboxymethyl cellulose (CMC)	Calcined colemanite	Compared with traditional bentonite production particles, its performance was superior.	Sivrikaya et al. [45]
Carboxymethyl cellulose (CMC)	Bentonite	CMC increased the hydrophilicity of bentonite and significantly improved green pellet performance.	Li et al. [46]
Carboxymethyl cellulose (CMC)	Nano-CaCO_3_	The compressive strength of both preheated pellets and sintered pellets exceeded industrial standards.	Lu et al. [47]
Carboxymethyl starch (CMS)	/	Drop strength and compression strengths of dry pellets exceeded industry standards.	Lu et al. [48]
Carboxymethyl starch (CMS)	Nano-CaCO_3_	CMS and nano-CaCO_3_ had high binding capacity and a higher adsorption rate.	Yuan et al. [49]
Carboxymethyl starch (CMS)	Nano-CaCO_3_	CCMS (nano-CaCO_3_ and CMS) pellets had better preheating and sintering strength, and the total iron content increased by 1.04%.	Lu et al. [50]

**Table 2:** LD sludge prevents strength deterioration [39].

Temperature (°C)	100	200	250	300	350	400	500	700
Compressive strength of bentonite (kg/pellet)	7.19	7.70	7.98	7.88	6.36	7.31	8.00	9.28
Compressive strength of 0.2% Ca-LS (kg/pellet)	5.91	6.90	6.91	6.48	5.47	6.61	7.99	8.31
Compressive strength of 0.2% Ca-LS+3% LDS (kg/pellet)	5.85	6.08	6.21	5.20	4.42	4.66	7.18	7.78

**Table 3:** Physicochemical properties of pellets.

Sample	Fe_total_	RI, %	RDI, %	Swelling Index, %	Ref.
Bentonite	60.93	65.6	3.4	7.4	Pal et al. [39]
0.3% Ca-LS + 5% LDS	62.53	64.5	1.5	6.8	
0.4% Ca-LS + 5% LDS	63.13	66.1	1.8	5.8	
Bentonite	65.22	77.06	18.5	9.09	Ammasi et al. [42]
0.5% Na-LS	\	75.93	27.17	13.09	
0.5% Na-LS + 1% Cu-SS	65.7	82.9	17.97	10.11	

## Data Availability

No new data were created or analyzed in this study. Data sharing is not applicable to this article.

## References

[1] Ma L.M. (2025). Study on the Liquid Phase Formation and Crystallization Mechanism of Medium-High Silica Fluxed Pellets.

[2] Zhou X.L., Zhen Z.K., Wan J.Y., Chen T.J., Luo Y.H., Wang Z.C. (2025). Study on the consolidation mechanism of organic binders high-grade pellets. Sinter. Pelletizing.

[3] Xu C.Y. (2022). Research on Reduction Behavior of Fluxed Pellets Under Hydrogen-Rich Conditions in Blast Furnace.

[4] Wang X.D., Jin Y.L. (2021). Strategy analysis and testing study of high ratio of pellet utilized in blast furnace. Iron Steel.

[5] Eisele T.C., Kawatra S.K. (2003). A review of binders in iron ore pelletization. Miner. Process. Extr. Metall. Rev..

[6] Cassola M.S., Chaves A.P. (1998). Effect of the addition of organic binders on the behavior of iron ore pellets. KONA Powder Part. J..

[7] Halt J.A., Kawatra S.K. (2014). Review of organic binders for iron ore concentrate agglomeration. Min. Metall. Explor..

[8] Sivrikaya O., Arol A.I. (2010). Use of boron compounds as binders in iron ore pelletization. Open Miner. Process. J..

[9] Kawatra S.K., Claremboux V. (2022). Iron ore pelletization: Part II. Inorg. binders. Miner. Process. Extr. Metall. Rev..

[10] Hayati-Ashtiani M., Jazayeri S.H., Ghannadi M., Nozad A. (2011). Experimental characterizations and swelling studies of natural and activated bentonites with their commercial applications. J. Chem. Eng. Jpn..

[11] Devasahayam S. (2018). A novel iron ore pelletization for increased strength under ambient conditions. Sustain. Mater. Technol..

[12] Zhao H.X., Zhou F.S., Zhao H.Y., Ma C.F., Zhou Y. (2022). A review on the effect of the mechanism of organic polymers on pellet properties for iron ore beneficiation. Polymers.

[13] Zhao H.X., Zhou F.S., Ma C.F., Wei Z.J., Long W.J. (2022). Bonding mechanism and process characteristics of special polymers applied in pelletizing binders. Coatings.

[14] Mendoza S., Yin B.H., Zhang A., Bumby C.W. (2022). Pelletization and sintering of New Zealand titanomagnetite ironsand. Adv. Powder Technol..

[15] Claremboux V., Kawatra S.K. (2023). Iron ore pelletization: Part III. organic binders. Miner. Process. Extr. Metall. Rev..

[16] Dronnet V.M., Renard C., Axelos M.A.V., Thibault J.-F. (1997). Binding of divalent metal cations by sugar-beet pulp. Carbohydr. Polym..

[17] Tuisov A.G., Kychkin A., Kychkin A.K., Anan’eva E.S. (2023). Reinforced Epoxy Binder Modified with Borpolymer. Polymers.

[18] Halt J.A., Kawatra S.K. (2017). Does Zeta Potential Iron Ore Conc. Affect Strength Dustiness Unfired Fired Pellets?. Miner. Process. Extr. Metall. Rev..

[19] De Moraes S.L., Lima J.R.B., Neto J.B.F. (2018). Effect of colloidal agents in iron ore pelletizing. Miner. Process. Extr. Metall. Rev..

[20] Lyons R.G., Kundrat D.M., Myers J.C., Wise D. (1986). Evaluation of taconite pellets made with an organic binder. Ironmak. Proc..

[21] De Moraes S.L., Lima J.R.B., Neto J.B.F., Fredericci C., Saccoccio E.M. (2020). Binding mechanism in green iron ore pellets with an organic binder. Miner. Process. Extr. Metall. Rev..

[22] Han G., Zhang Y., Huang Y., Sun Z., Li G. (2011). Effects of Binders on Oxidized Pellets Preparation from Vanadium/Titanium-Bearing Magnetite. 2nd International Symposium on High-Temperature Metallurgical Processing.

[23] Zhang S.H., Shao J.N., Lan C.C., Bi Z.X., Lv Q. (2022). Application status and prospect of biomass energy in ironmaking process. Iron Steel.

[24] Fu P., Xu G.P., Li X.H., Zhu T., Zhang Z. (2021). Analysis of the Development Status and Trends of China’s Biomass Power Industry and Carbon Emission Reduction Potential. Ind. Saf. Environ. Prot..

[25] Garcia R., Gil M.V., Rubiera F., Pevida C. (2019). Pelletization of wood and alternative residual biomass blends for producing industrial quality pellets Science. Fuel.

[26] Klemm D., Heublein B., Fink-habil H.P., Bohn A. (2005). Cellulose, chemistry and application. Angew. Chem. Int. Ed..

[27] Zhao Z., Hao S., Hao P., Song Y.H., Manivannan A., Wu N., Liu H. (2015). Lignosulphonate-cellulose derived porous activated carbon for supercapacitor electrode. J. Mater. Chem. A.

[28] Watcharakitti J., Win E.E., Nimnuan J., Smith S.M. (2022). Modified starch-based adhesives: A review. Polymers.

[29] Qiu G.Z., Jiang T.J., Li H.X., Wang D.Z. (2003). Functions and molecular structure of organic binders for iron ore pelletization. Colloids Surf. A Physicochem. Eng. Asp..

[30] Li H.X., Jang T., Qiu G.Z., Wang D.Z. (2000). Molecular Structure Mould and Selecting Criterion of Organic Binder for Iron Ore Pellet.

[31] Han G.H., Huang Y.F., Li G.H., Zhang Y.B., Zhou Y.L., Jiang T. (2012). Optimizing the mass ratio of two organic active fractions in modified humic acid (MHA) binders for iron ore pelletizing. ISIJ Int..

[32] Zhu D., Pan J., Lu L., Holmes R.J. (2015). Iron Ore: Mineralogy, Processing and Environmental Sustainability.

[33] Qiu G.Z., Jiang T., Fan X.H., Zhu D.Q., Huang Z.C. (2004). Effects of binders on balling behaviors of iron ore concentrates. Scand. J. Metall..

[34] Vermeer A.W.P., Van Riemsdijk W.H., Koopal L.K. (1998). Adsorption of humic acid to mineral particles. 1. Specific and electrostatic interactions. Langmuir.

[35] Gu B.H., Schmitt J., Chen Z.H., Liang L.Y., McCarthy J.F. (1994). Adsorption and desorption of natural organic matter on iron oxide: Mechanisms and models. Environ. Sci. Technol..

[36] Zhang Y.B., Li P., Zhou Y.L., Han G.H., Li G.H., Xu B., Jiang T. (2012). Adsorption of lignite humic acid onto magnetite particle surface. J. Cent. South Univ..

[37] Casey L. (2016). Organic Binders for Iron Ore Pelletization. Master’s Thesis.

[38] Forsmo S. (2007). Influence of Green Pellet Properties on Pelletizing of Magnetite Iron Ore. Ph.D. Thesis.

[39] Pal J., Ammasi A., Bandyopadhyay B.S., Dwarapudi S., Paul I. (2024). An innovative approach to replace bentonite in hematite ore pelletizing with organic binder. Miner. Process. Extr. Metall. Rev..

[40] Fang H.Y., Gao L., Zhou X.L., Yan H.L., Wang Y.P., Ji H.H. (2023). Enhanced compressive strength of preheated limonite pellets with biomass-derived binders. Adv. Powder Technol..

[41] Tafadzwa N., Mavengere S., Bright S., Mapamba L. (2023). Investigating the effectiveness of organic binders as an alternative to bentonite in the pelletization of low-grade iron ore. Physicochem. Probl. Miner. Process..

[42] Ammasi A., Pal J. (2016). Replacement of bentonite in hematite ore pelletisation using a combination of sodium lignosulphonate and copper smelting slag. Ironmak. Steelmak..

[43] Zhou J.A., Wang J., Wang B., Ding B., Dang Y.C., Li Y.J. (2022). The bonding mechanism and effects of sodium lignosulfonate (SL) in iron ore pelletization. Metall. Res. Technol..

[44] Parathodiel H., Mousa E., Ahmed H., Elsadek M., Forsberg K., Andersson C. (2023). Developing iron ore pellets using novel binders for H_2_-based direct reduction. Sustainability.

[45] Sivrikaya O., Arol A.I. Use of organic binders and borates in pelletizing of iron oxides. Proceedings of the 4th International Boron Symposium.

[46] Li C.X., Bai Y., Ren R.C., Liu G.Q., Zhao J.Y. (2019). Study of the mechanism for improving green pellet performance with compound binders. Physicochem. Probl. Miner. Process..

[47] Lu J.W., Zhao X., Yuan Z.T., Gao P.C., Li L.X. (2019). Characterization of the Bonding Effect of Nano-CaCO_3_ Modified CMC on Magnetite Concentrate Pellets. J. Miner. Met. Mater. Soc..

[48] Lu S., Yuan Z., Zhang C. (2018). Binding mechanisms of polysaccharides adsorbing onto magnetite concentrate surface. Powder Technol..

[49] Yuan Z.T., Lu S.S., Liu J.T., Gao P.C., Lu J.W., Li L.X. (2017). Effects of nano-CaCO_3_ on the adsorption of carboxymethyl starch onto magnetite concentrate in pelletizing. Powder Technol..

[50] Lu S.S., Yuan Z.T., Liu J.T., Lu J.W., Li L.X., Hao H.Q. (2016). Binding effects and mechanisms of the carboxymethyl starch modified with nano-CaCO_3_ in magnetite concentrate pellets. Powder Technol..

[51] Meister J.J. (2002). Modification of lignin. J. Macromol. Sci..

[52] Gonçalves S., Ferra J., Paiva N., Martins J., Carvalho L.H., Magalhães F.D. (2021). Lignosulphonates as an alternative to non-renewable binders in wood-based materials. Polymers.

[53] Aro T., Fatehi P. (2017). Production and application of lignosulfonates and sulfonated lignin. ChemSusChem.

[54] Sixta H. (2006). Handbook of Pulp, 2 Volume Set.

[55] Suhr M., Klein G., Kourti I., Gonzalo M.R., Santonja G.G., Roudier S., Sancho L.D. (2015). Best available techniques (BAT) reference document for the production of pulp, paper and board. Eur. Comm..

[56] Northey R.A. (2002). The use of lignosulfonates as water reducing agents in the manufacture of gypsum wallboard. Chemical Modification, Properties, and Usage of Lignin.

[57] Stern T., Schwarzbauer P. (2008). Wood-based lignosulfonate versus synthetic polycarboxylate in concrete admixture systems: The perspective of a traditional pulping by-product competing with an oil-based substitute in a business-to-business market in central Europe. For. Prod. J..

[58] Myrvold B.O. (2008). A new model for the structure of lignosulphonates: Part 1. Behaviour in dilute solutions. Ind. Crops Prod..

[59] Salazar Valencia P.J., Bolívar Marinez L.E., Pérez Merchancano S.T. (2015). Molecular modeling of ammonium, calcium, sulfur, and sodium lignosulphonates in acid and basic aqueous environments. Braz. J. Phys..

[60] Kun D., Pukánszky B. (2017). Polymer/lignin blends: Interactions, properties, applications. Eur. Polym. J..

[61] Fiorani G., Crestini C., Selva M., Perosa A. (2020). Advancements and complexities in the conversion of lignocellulose into chemicals and materials. Front. Chem..

[62] Liu Q., Li X., Tang J., Zhou Y., Lin Q., Xiao R., Zhang M. (2019). Characterization of goethite-fulvic acid composites and their impact on the immobility of Pb/Cd in soil. Chemosphere.

[63] Li Q., Zeng M.J., Zhu D.M., Lou H.M., Pang Y.X., Qiu K.X., Huang J.H., Qiu X.Q. (2019). A simple and rapid method to determine sulfonation degree of lignosulfonates. BioEnergy Res..

[64] Duval A., Molina-Boisseau S., Chirat C. (2015). Fractionation of lignosulfonates: Comparison of ultrafiltration and ethanol solubility to obtain a set of fractions with distinct properties. Holzforschung.

[65] Lou H.M., Lai H.R., Wang M.X., Pang Y.X., Yang D.J., Qiu X.Q., Wang B., Zhang H.B. (2013). Preparation of lignin-based superplasticizer by graft sulfonation and investigation of the dispersive performance and mechanism in a cementitious system. Ind. Eng. Chem. Res..

[66] Fredheim G.E., Braaten S.M., Christensen B.E. (2002). Molecular weight determination of lignosulfonates by size-exclusion chromatography and multi-angle laser light scattering. J. Chromatogr. A.

[67] Buchholz R.F., Neal J.A., McCarthy J.L. (1992). Some properties of paucidisperse gymnosperm lignin sulfonates of different molecular weights. J. Wood Chem. Technol..

[68] Fan J.J., Qiu G.Z., Jiang T., Guo Y.F., Hao H.Z., Yang Y.B. (2012). Mechanism of high pressure roll grinding on compression strength of oxidized hematite pellets. J. Cent. South Univ..

[69] Kukrety A., Singh R.K., Singh P., Ray S.S. (2018). Comprehension on the synthesis of carboxymethylcellulose (CMC) utilizing various cellulose rich waste biomass resources. Waste Biomass Valorization.

[70] Slotte S. (2021). Production Process of Carboxymethyl Cellulose. Bachelor’s Thesis.

[71] Rahman M.S., Hasan M.S., Nitai A.S., Nam S., Karmakar A.K., Ahsan M.S., Shiddiky M.J.A., Ahmed M.B. (2021). Recent developments of carboxymethyl cellulose. Polymers.

[72] Aggrey W.N., Asiedu N.Y., Tackie-Otoo B.N., Adjei S., Mensah-Bonsu E. (2019). Performance of carboxymethyl cellulose produced from cocoa pod husk as fluid loss control agent at high temperatures and variable (low and high) differential pressure conditions-Part 1. J. Pet. Sci. Technol..

[73] Baruah J., Nath B.K., Sharma R., Kumar S., Deka R.C., Baruah D.C., Kalita E. (2018). Recent trends in the pretreatment of lignocellulosic biomass for value-added products. Front. Energy Res..

[74] Kopania E., Wietecha J., Ciechańska D. (2012). Studies on isolation of cellulose fibres from waste plant biomass. Fibres Text. East. Eur..

[75] Michelin M., Gomes D.G., Romaní A., Polizeli M.D.L.T., Teixeira J.A. (2020). Nanocellulose production: Exploring the enzymatic route and residues of pulp and paper industry. Molecules.

[76] Abdel Ghaffar A.M., El-Arnaouty M.B., Abdel Baky A.A., Shama S.A. (2016). Radiation-induced grafting of acrylamide and methacrylic acid individually onto carboxymethyl cellulose for removal of hazardous water pollutants. Des. Monomers Polym..

[77] El-Sakhawy M., Kamel S., Salama A., Sarhan H.A. (2014). Carboxymethyl cellulose acetate butyrate: A review of the preparations, properties, and applications. J. Drug Deliv..

[78] Kunjalukkal Padmanabhan S., Lamanna L., Friuli M., Sannino A., Demitri C., Licciulli A. (2023). Carboxymethylcellulose-based hydrogel obtained from bacterial cellulose. Molecules.

[79] Yaradoddi J.S., Banapurmath N.R., Ganachari S.V., Soudagar M.E.M., Mubarak N.M., Hallad S., Hugar S., Fayaz H. (2020). Biodegradable carboxymethyl cellulose based material for sustainable packaging application. Sci. Rep..

[80] Hashem A., Farag S., Badawy S.M. (2025). Carboxymethyl cellulose: Past innovations, present applications, and future horizons. Results Chem..

[81] Pinto E., Aggrey W.N., Boakye P., Amenuvor G., Sokama-Neuyam Y.A., Fokuo M.K., Karimaie H., Sarkodie K., Adenutsi C.D., Erzuah S. (2022). Cellulose processing from biomass and its derivatization into carboxymethylcellulose: A review. Sci. Afr..

[82] Hoogendam C.W., De Keizer A., Cohen Stuart M.A., Bijsterbosch B.H., Batelaan J.G., Van der Horst P.M. (1998). Adsorption mechanisms of carboxymethyl cellulose on mineral surfaces. Langmuir.

[83] Saha B., Patra A.S., Mukherjee A.K., Paul I. (2021). Interaction and thermal stability of carboxymethyl cellulose on α-Fe_2_O_3_ (001) surface: ReaxFF molecular dynamics simulations study. J. Mol. Graph. Model..

[84] Zhivkov A.M. (2013). Electric properties of carboxymethyl cellulose. Cellulose-Fundamental Aspects.

[85] Jalil R., Sarif M., Elham P., Hashim S. (2022). Synthesis of carboxymethyl cellulose (CMC) from delignified Dyera Costulata. Malays. J. Chem. Eng. Technol..

[86] Casaburi A., Rojo Ú.M., Cerrutti P., Vázquez A., Foresti M.L. (2018). Carboxymethyl cellulose with tailored degree of substitution obtained from bacterial cellulose. Food Hydrocoll..

[87] Yang G., Fan X., Chen X., Yuan L.S., Huang X.X., Li X. (2015). Interaction mechanism between carboxymethyl cellulose and iron ore concentrates in iron ore agglomeration. J. Cent. South Univ..

[88] Li D.G. (2008). Properties of sodium carboxymethyl cellulose and its application in papermaking industry. Heilongjiang Pap. Mak..

[89] Fan X.H., Yang G.M., Chen X.L., He X.N., Huang X.X., Gao L. (2014). Effect of carboxymethyl cellulose on the drying dynamics and thermal cracking performance of iron ore green pellets. Powder Technol..

[90] De Britto D., Assis O.B.G. (2009). Thermal degradation of carboxymethylcellulose in different salty forms. Thermochim. Acta.

[91] Srivastava U., Kawatra S.K., Eisele T.C. (2013). Study of organic and inorganic binders on strength of iron oxide pellets. Metall. Mater. Trans. B.

[92] Dwarapudi S., Ghosh T.K., Shankar A., Tathavadkar V., Bhattacharjee D., Venugopal R. (2011). Effect of pellet basicity and MgO content on the quality and microstructure of hematite pellets. Int. J. Miner. Process..

[93] Copeland L., Blazek J., Salman H., Tang M.C. (2009). Form and functionality of starch. Food Hydrocoll..

[94] Bertoft E. (2017). Understanding starch structure: Recent progress. Agronomy.

[95] Seidel C., Kulicke W.H., Heß C., Hartmann B., Lechner M.D., Lazik W. (2004). Synthesis and characterization of cross-linked carboxymethyl potato starch ether gels. Starch-Stärke.

[96] Spychaj T., Wilpiszewska K., Zdanowicz M. (2013). Medium and high substituted carboxymethyl starch: Synthesis, characterization and application. Starch-Stärke.

[97] Heinze T., Pfeiffer K., Liebert T., Heinze U. (1999). Effective approaches for estimating the functionalization pattern of carboxymethyl starch of different origin. Starch-Stärke.

[98] Stojanović Ž., Jeremić K., Jovanović S. (2000). Synthesis of carboxymethyl starch. Starch-Stärke.

[99] Thomson R.A.M. (1983). Chemistry and Technology of Water-Soluble Polymers.

[100] Sidar A., Albuquerque E.D., Voshol G.P., Ram A.F., Vijgenboom E., Punt P.J. (2020). Carbohydrate binding modules: Diversity of domain architecture in amylases and cellulases from filamentous microorganisms. Front. Bioeng. Biotechnol..

[101] Lemieux M., Gosselin P., Mateescu M.A. (2010). Influence of drying procedure and of low degree of substitution on the structural and drug release properties of carboxymethyl starch. AAPS PharmSciTech.

[102] Li H.X., Wang D.Z., Hu Y.H., Qiu G.Z., Jiang T. (2001). The mechanism of improving pellet strength by carboxyl methlatedamylum. Cent. South Univ. Technol. (Dep. Miner. Eng.).

